# Inhibition of calcium/calmodulin-dependent kinase II restores contraction and relaxation in isolated cardiac muscle from type 2 diabetic rats

**DOI:** 10.1186/s12933-018-0732-x

**Published:** 2018-06-14

**Authors:** Lorna J. Daniels, Rachel S. Wallace, Olivia M. Nicholson, Genevieve A. Wilson, Fiona J. McDonald, Peter P. Jones, J. Chris Baldi, Regis R. Lamberts, Jeffrey R. Erickson

**Affiliations:** 1 Otago School of Medical Sciences, Department of Physiology and HeartOtago, University of Otago, 270 Great King Street, Dunedin, 9016 New Zealand; 2 Otago School of Medical Sciences, Department of Medicine and HeartOtago, University of Otago, Dunedin, New Zealand

**Keywords:** Calcium/calmodulin-dependent kinase II, Diabetes mellitus, Diabetic cardiac dysfunction, Echocardiography, Cardiac contractility

## Abstract

**Background:**

Calcium/calmodulin-dependent kinase II-delta (CaMKIIδ) activity is enhanced during hyperglycemia and has been shown to alter intracellular calcium handling in cardiomyocytes, ultimately leading to reduced cardiac performance. However, the effects of CaMKIIδ on cardiac contractility during type 2 diabetes are undefined.

**Methods:**

We examined the expression and activation of CaMKIIδ in right atrial appendages from non-diabetic and type 2 diabetic patients (n = 7 patients per group) with preserved ejection fraction, and also in right ventricular tissue from Zucker Diabetic Fatty rats (ZDF) (n = 5–10 animals per group) during early diabetic cardiac dysfunction, using immunoblot. We also measured whole heart function of ZDF and control rats using echocardiography. Then we measured contraction and relaxation parameters of isolated trabeculae from ZDF to control rats in the presence and absence of CaMKII inhibitors.

**Results:**

CaMKIIδ phosphorylation (at Thr287) was increased in both the diabetic human and animal tissue, indicating increased CaMKIIδ activation in the type 2 diabetic heart. Basal cardiac contractility and relaxation were impaired in the cardiac muscles from the diabetic rats, and CaMKII inhibition with KN93 partially restored contractility and relaxation. Autocamtide-2-related-inhibitor peptide (AIP), another CaMKII inhibitor that acts via a different mechanism than KN93, fully restored cardiac contractility and relaxation.

**Conclusions:**

Our results indicate that CaMKIIδ plays a key role in modulating performance of the diabetic heart, and moreover, suggest a potential therapeutic role for CaMKII inhibitors in improving myocardial function during type 2 diabetes.

## Background

Altered calcium (Ca^2+^) handling has been identified as a key contributor to diabetic cardiac dysfunction (DCD) [1, 2]. Ca^2+^ is a critical second messenger in cardiac muscle, and thus changes in Ca^2+^ handling have acute and chronic consequences on cardiac function [3, 4]. Increased resting intracellular Ca^2+^ levels, attenuated sarcoplasmic reticulum Ca^2+^ release and re-uptake, and delayed recovery of the intracellular Ca^2+^ transient have been observed in rodent models of both type 1 and type 2 diabetes, ultimately resulting in reduced contractility and relaxation of cardiac muscles [5–8]. However, the key mechanisms underpinning perturbations of contractility in the diabetic heart remain to be determined.

One emerging mediator of cardiac contractility is the myocardial isoform of Ca^2+^/calmodulin-dependent protein kinase II (CaMKIIδ) [9–11]. This multifunctional serine/threonine protein kinase regulates proteins associated with cardiac Ca^2+^ flux, including ryanodine receptors [12], phospholamban [13], and L-type Ca^2+^ channels [14], as well as proteins integral to sarcomere structure [15] and cross bridge cycling [16]. Therefore, CaMKIIδ plays a pivotal role in cardiac contraction and relaxation. Persistent activation of CaMKIIδ is linked to a number of cardiac pathologies, including maladaptive hypertrophy [17, 18] apoptosis [19, 20] and arrhythmogenic events [21, 22].

Inhibition of CaMKIIδ has emerged as a potential therapeutic strategy for the prevention of cardiac dysfunction [23]. Previous studies using non-diabetic animal models with global deletion of CaMKIIδ have reported protection against cardiac hypertrophy [24], apoptosis [25], and Ca^2+^ leak induced arrhythmia [26]. Pharmacological inhibition of CaMKIIδ has also shown promising results both in vitro and in vivo. For example, acute CaMKIIδ inhibition in isolated cardiac muscle taken from non-diabetic patients with ischemic heart failure resulted in increased contractility and reduced Ca^2+^ spark frequency [27]. Recent studies have demonstrated that CaMKIIδ activity is increased in myocytes subjected to hyperglycemia or myocytes isolated from animal models of type 1 diabetes [28, 29]. However, the effects of CaMKIIδ inhibition on cardiac function in type 2 diabetes, which comprises about 95% of diabetes diagnoses in human patients, have not been determined.

Thus, the present study examined the link between CaMKIIδ activation and cardiac contractile function in a rat model of type 2 diabetes with preserved ejection fraction (EF). We found that the phosphorylated form of CaMKIIδ was upregulated in the hearts of patients with type 2 diabetes, even prior to the development of altered heart function. We then used isolated ventricular trabeculae from non-diabetic (nDM) and type 2 diabetic (DM) Zucker Diabetic Fatty (ZDF) rats to measure contraction and relaxation properties of the cardiac muscle with and without CaMKIIδ inhibitors. Our data demonstrate that CaMKIIδ inhibition attenuated the reduced force development and impaired rates of contraction and relaxation associated with type 2 diabetes, and moreover that these effects are independent of Ca^2+^ flux properties, including transient amplitude and Ca^2+^ load. These findings extend our understanding of the role of CaMKIIδ in regulating the function of the heart in the context of diabetes and suggest a novel therapeutic potential for CaMKIIδ inhibition to reverse impaired contractility and prevent heart failure progression in diabetic patients.

## Research design and methods

### Human tissue acquisition and echocardiography

Human right atrial tissue samples were collected from seven non-diabetic and seven diabetic patients undergoing on-pump coronary artery bypass graft surgery after written consent. Our informed consent practice conformed to the principles outlined in the Declaration of Helsinki and was approved by the Human and Disability Ethics Committee of New Zealand (LRS/12/01/001). To prevent systolic heart failure from acting as a confounding factor, only patients with an (EF) over 55% were included in the study. No other exclusion criteria were used. Table 1 shows summary statistics for the nDM and diabetic (DM) groups, including gender, age, etc.

**Table 1:** Diabetic (n = 7) and non-diabetic (n = 7) patient characteristics and echocardiographic data

Parameter	Non-diabetic patient	Type 2 diabetic patient	p-value
Age (years)	68.0 ± 2.0	65.2 ± 2.6	0.41
Sex	6 M, 1 F	6 M, 1 F	n/a
BMI	28.3 ± 1.6	32.6 ± 2.4	0.14
HbA1C (%)	5.4 ± 0.2	7.4 ± 0.5	0.01*
HbA1C (mmol/mol)	36 ± 2	58 ± 6	
Glucose (mmol/L)	5.9 ± 0.3	8.1 ± 1.6	0.20
Duration of diabetes (y)	12.2 ± 2.3	n/a	n/a
MAP (mmHg)	98.4 ± 5.3	n/a	0.91
Beta blockers	4/7	6/7	n/a
ACE inhibitors	6/7	5/7	n/a
LVEDV (mL)	88 ± 12	86 ± 8	0.89
LVESV (mL)	36 ± 6	35 ± 4	0.92
EF (%)	60 ± 2	60 ± 1	0.86
E vel (cm/s)	0.6 ± 0.1	0.7 ± 0.1	0.56
A vel (cm/s)	0.8 ± 0.1	0.9 ± 0.1	0.26
E/A	0.9 ± 0.1	0.8 ± 0.1	0.50

Data are expressed ± SEM

*M* male, *F* female, *HbA1C* glycated haemoglobin, *MAP* mean arterial blood pressure, *LVEDV* left ventricular end diastolic diameter, *LVESV* left ventricular end systolic diameter, *EF* ejection fraction

For all parameters independent t-test, * p < 0.05

In all patients, right atrial appendages (RAA), cardiac tissue that lies anterior and medial of the right atrium, were removed under normothermic conditions before cross clamping for cardiopulmonary bypass. Immediately after removal, all specimens were placed in a sealed vial containing modified, low Ca^2+^ (0.5 mM) Krebs–Henseleit buffer ((mM): 118.5 NaCl, 4.5 KCl, 0.3 NaH_2_PO4, 1.0 MgCl_2_6H_2_O, 25 NaHCO_3_ and 11 glucose). Within 5–10 min after removal a piece of the RAA was flash-frozen and stored at − 80 °C.

Echocardiographic examinations were performed using a Vivid E9 (GE Medical systems, Milwaukee, WI, USA) ultrasound system. All images were obtained by a trained sonographer using conventional echocardiographic patient positioning. Left ventricular volumes at end-diastole (LVEDV) and end-systole (LVESV) were obtained in the apical four and two chamber views. Peak early diastolic filling velocity (E) and late diastolic filling velocity (A) were obtained in the apical four chamber view using pulsed wave Doppler with the sample volume placed between the mitral valve leaflets [30]. Volumes were visually traced with papillary muscles excluded and calculated using the modified Simpson’s biplane method in accordance with ASE guidelines [31]. EF was derived using two-dimensional echocardiography.

### Animals

All procedures were approved by the University of Otago Animal Ethics Committee and were conducted in accordance with the New Zealand Animal Welfare Act (1999) and the NIH Guide for the Care and Use of Laboratory Animals and approved by the Institutional Animal Care and Use Committee of the University of California, Davis. Experiments were performed with myocardial tissue from ZDF rats, which is a well-established model of type 2 diabetes mellitus [32]. ZDF rats with the homozygous missense mutation in the leptin receptor gene have impaired satiety signaling and hyperphagia, and develop diabetes from 12 weeks of age due to impaired pancreatic beta-cell function. Lean non-diabetic littermates were used for comparison as in-strain controls. Male ZDF rats were housed at 20 ± 1 °C under a 12 h light–dark cycle and provided with food and water ad libitum. All animals were maintained on a Purina 5008 diet (LabDiet, St. Louis, MO, USA). Blood glucose measurements were taken at 12 and 20 weeks of age via tail vein blood using a glucometer (Roche, Basel, Switzerland), and bodyweight was recorded.

### Animal echocardiography

Echocardiography was carried out at 12 and 20 weeks of age. Animals were maintained under isoflurane at 3% and standard two-dimensional echocardiographic left ventricular parameters were obtained from the parasternal short and long axis. All settings were optimized to obtain maximal signal-to-noise ratio and two-dimensional images to provide optimal endocardial delineation. All echocardiography data was independently analyzed by two blinded researchers, and their results were compared to control for potential variation in analysis.

### Protein analysis

Right ventricular (RV) tissue from nDM to DM ZDF rats and RAA tissue from human patients were homogenized in buffer containing: 50 mM Tris–HCl, pH 7.5, 3% SDS, phenyl methyl sulfonyl fluoride (PMSF) and phosphatase inhibitor (Roche). Cardiac tissue homogenates were separated on 10% SDS–polyacrylamide gels and blotted using primary antibodies against total CaMKIIδ (1:3000, Thermofisher Scientific PA5-22168), Thr287 phosphorylated CaMKIIδ (1:1000, Abcam AB32678), and GAPDH (1:10,000, Genetex GTX627408). Horseradish peroxidase-conjugated (HRP) secondary antibodies (1:2500, Thermofisher Scientific 31460, 31430) against rabbit and mouse primary antibodies were subsequently used. Chemiluminescent detection was performed with Supersignal west-pico (Millipore) and imaged using a Syngene gel doc system. Total and phosphorylated CaMKIIδ band intensities were normalized to GAPDH. Ratios are presented as phosphorylated CaMKIIδ relative to total CaMKIIδ, as a measure of CaMKIIδ activity.

### Trabeculae preparation and experiments

Once the echocardiographic examination had been completed, the animals were allowed to recover for 1 h before being sacrificed. The hearts were removed under anesthesia (pentobarbital; 60 mg/kg) and placed in a modified Krebs-Henseleit buffer (KHB) (mM: 118.5 NaCl, 1 CaCl_2_, 0.33 NaH_2_PO_4_, 1.0 MgCl_2_6H_2_O, 25 NaHCO_3_ and 11 glucose) with 14 mM KCl oxygenated with carbogen (95% O_2_–5% CO_2_). The heart was then mounted on a modified Langendorff setup and retrograde perfused via the aorta with modified KHB. The RV was opened and a suitable cardiac muscle (trabeculae) (dimensions: Length 3.6 ± 0.2 mm, Width 0.5 ± 0.1 mm, Depth 0.2 ± 0.1 mm) was dissected under a microscope. Trabeculae were then transferred to the experimental chamber and attached to a force transducer and micromanipulator. The muscles were continuously superfused with modified oxygenated KHB at 37 °C and stimulated at a basal stimulation frequency of 2 Hz.

After an equilibration period of 20 min, the trabeculae were gradually stretched to muscle length to achieve maximal isometric force development. Once a steady-state twitch force was achieved, stimulation was stopped and trabeculae were superfused for 20 min with KN93 (2 μM) or with the inactive analogue KN92 (2 μM). Additional experiments were carried out using a myristoylated, cell permeant form of autocamtide-2-related-inhibitor peptide (AIP, 2.5 μM), with KHB used as a control. Force frequency relationships were obtained by measuring steady-state twitch force conditions at stimulation frequencies of 2, 3, 4, 5 and 6 Hz. No animal was excluded from the study, but a small number of trabeculae showed negative force-frequency relationships in the contractility measurements, a sign of poor muscle quality, and were excluded.

### Isolation of ZDF rat ventricular myocytes

Cardiac ventricular myocytes were isolated from the ventricles of 20 week old male nDM and DM ZDF rats. Animals were anti-coagulated with intraperitoneal injection of heparin (400 mg/kg), followed by anaesthesia in a gas chamber with 5% isoflurane (100% oxygen). Hearts were removed, cannulated and subjected to retrograde aortic perfusion at 37 °C at a rate of 12–14 mL/min. Hearts were perfused in Ca^2+^ free buffer for 10 min followed by perfusion for 12 min with Minimum Essential Media (Thermofisher). Hearts were removed from the cannula and the ventricle was dissociated at room temperature by pipetting gently up and down. The cell suspension was filtered and the collagenase inactivated by re-suspending the tissue in medium containing 10% bovine calf serum. Calcium was gradually added back to a final concentration of 1 mM.

### Measurement of Ca^2+^ transients and sarcoplasmic Ca^2+^ load

Isolated ventricular cardiomyocytes were incubated on laminin-coated cover glass slides and loaded with Ca^2+^-fluorescent dye Fluo-4AM (Molecular Probes) for 25 min. To wash out extracellular dye, the Fluo-4AM solution was removed from the cover glass slides and cells incubated in Tyrode’s solution (mM: 140 NaCl, 4 KCl, 1.1 MgCl_2_, 10 HEPES, 10 glucose, 1.8 CaCl2; pH = 7.4, with NaOH) either containing KN92 (1 µM, Millipore), KN93 (1 µM, Millipore), AIP (1 µM, Tocris Bioscience) or control solution (Tyrode’s solution) for 10 min. Line scans were carried out on a confocal microscope (Bio Rad, Radiance 2100 × 40 oil immersion objective) in line scan mode (3 ms/line). Fluo-4AM was excited with an Argon laser (488 nm) and emission was collected at wavelengths > 505 nm. Ca^2+^ transients were evoked by field stimulation (1 Hz) using a Grass S48 stimulator. Sarcoplasmic reticulum (SR) Ca^2+^ load was assessed by rapid application of caffeine (10 mM, Sigma Aldrich) after 30 s to reach steady state pacing. During recording, intact myocytes were continuously perfused with either Tyrode’s solution for control experiments, or Tyrode’s solution containing KN92 (1 µM), KN93 (1 µM) or AIP (1 µM).

### Data analysis and statistics

GraphPad Prism (version 6.0) was used for all statistical analysis. One-way analysis of variance (ANOVA) was used for analysis of bodyweight, blood glucose and echocardiographic measurements, with p < 0.05 indicating statistical significance. An independent *t* test was used for comparison of CaMKIIδ expression and activation levels in human and animal tissue. Functional data was analyzed using Lab Chart 7.0 (Ad Instruments). Force values were normalized to the cross-sectional area of the trabeculae (width X thickness X π) and expressed in mN/mm^2^. Differences between nDM and DM ZDF rats and the effect of CaMKIIδ inhibition were analyzed using a two-way between groups ANOVA, with p < 0.05 indicating statistical significance. Linear regression was used for analysis of the relationship between the slopes of each parameter.

## Results

### CaMKIIδ modification is enhanced in cardiac tissue from diabetic patients

To assess CaMKIIδ expression and modification (as a measure of its activity) in the diabetic myocardium, we assembled cohorts of type 2 diabetic and non-diabetic patients with preserved EF and no clinical indication of heart failure. Summary data for the patient characteristics of each cohort are listed in Table 1. The diabetic patient cohort had mean diabetes duration of 12.2 ± 2.3 years, as well as significantly higher glycated hemoglobin (HbA1C) compared to the non-diabetic cohort as expected. There was no significant difference in glucose, age, mean arterial blood pressure, EF, LVESV, LVEDV, or peak early and late mitral inflow velocities between the type 2 diabetic and non-diabetic cohorts, indicating no evidence of heart failure in either cohort.

**Fig. 1:**
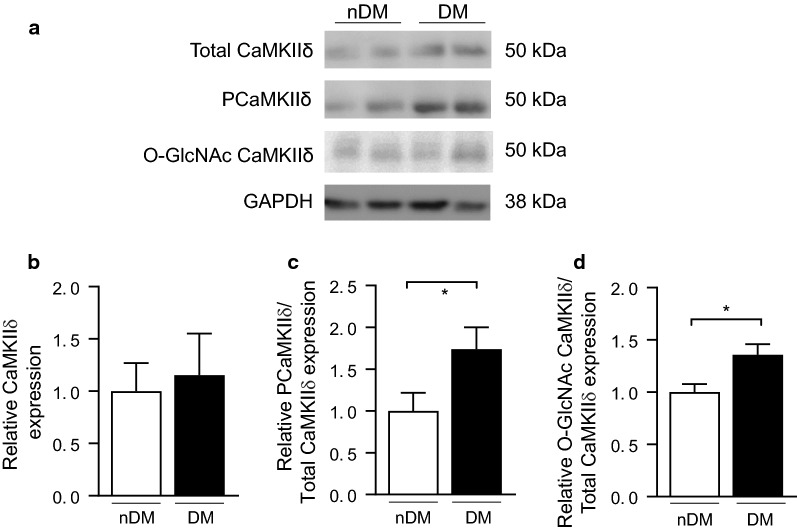
CaMKIIδ activation is increased in the right atrial appendage tissue from type 2 diabetic patients compared to non-diabetic patients. **a** Representative immunoblot of total CaMKIIδ, Thr287 phosphorylated (PCaMKIIδ) and GAPDH as a loading control in non-diabetic patients, and type 2 diabetic patients. **b** Quantification of total CaMKIIδ levels in non-diabetic patients (nDM) (n = 7) and type 2 diabetic patients (DM) (n = 7). **c** Quantification of PCaMKIIδ levels in non-diabetic patients (nDM) (n = 7) and type 2 diabetic patients (DM) (n = 7). **d** Quantification of O-GlcNAc modified CaMKIIδ levels in non-diabetic nDM and DM patients (DM) (n = 5). Data are mean ± SEM. *p < 0.05 for comparison of nDM and D values (unpaired t-test)

We hypothesized that CaMKIIδ modification would be increased in the myocardium of type 2 diabetic patients without heart failure CaMKIIδ modification is critically associated with activation of the kinase [33], and CaMKIIδ modification is known to be increased in diabetes [28, 29]. An example blot for total, phosphorylated, and *O*-GlcNAc modified CaMKIIδ in RAA tissue from our type 2 diabetic and non-diabetic cohorts is shown in Fig. 1a. There was no significant difference in total CaMKIIδ expression (1.00 ± 0.27 vs. 1.15 ± 0.40, non-diabetes vs. type 2 diabetes, Fig. 1b), but both phosphorylated (1.00 ± 0.22 vs. 1.74 ± 0.2, non-diabetes vs. type 2 diabetes, Fig. 1c) and *O*-GlcNAc modified (1.00 ± 0.12 vs. 1.40 ± 0.16, non-diabetes vs. type 2 diabetes, Fig. 1d) CaMKIIδ were significantly increased in the RAA tissue of type 2 diabetic patients compared to non-diabetic patients.

### CaMKIIδ phosphorylation is enhanced in a rat model of type 2 diabetes

To determine the functional role of CaMKIIδ in the diabetic heart, we first needed to assure that our animal model of diabetes had similar characteristics to our diabetic patient cohort. Table 2 summarizes our measurements of blood glucose, body weight, and heart function in the rats at 12 and 20 weeks. ZDF rats had significantly higher fasting blood glucose and body weight at both 12 and 20 weeks compared to lean controls. The increase in fasting blood glucose for the type 2 diabetic rats does differ from what we observed in our patient cohort, but this difference is likely a consequence of the human patients receiving blood glucose lowering treatment, while the animals received no such treatment. We found no significant difference in LVPWs, LVPWd, EF, LVESV, or peak early and late mitral inflow velocities between the nDM and DM ZDF rats at either time point. We did observe a significant increase in LVEDV in the DM ZDF rats; however, no effect was observed on the E/A ratio. We therefore concluded that there was no evidence of either diastolic or systolic heart failure in the DM rats at 20 weeks of age. Overall, our measurements indicate that the ZDF rats are diabetic at 20 weeks of age, but with preserved EF and no evidence of advanced heart failure.

**Table 2:** Bodyweight, blood glucose and echocardiographic assessment in non-diabetic (n = 10) and type 2 diabetic ZDF rats (n = 10)

Parameter	12 weeks		20 weeks		Condition effect (p value)
	nDM	DM	nDM	DM	
ZDF rats					
Bodyweight (g)	3.1 ± 0.1	375.3 ± 36.7	387.2 ± 7.9	442.9 ± 46.2	0.04*
Blood	8.6 ± 1.9	16.8 ± 2.5	8.2 ± 0.6	25.0 ± 2.5	0.01*
Glucose (mmol/L)					
LVPWs (mm)	3.1 ± 0.1	3.1 ± 0.1	2.9 ± 0.1	3.0 ± 0.1	0.60
LVPWd (mm)	2.5 ± 0.1	2.5 ± 0.2	2.3 ± 0.1	2.7 ± 0.1	0.18
EF (%)	77.2 ± 1.9	78.6 ± 1.2	72.2 ± 1.9	69.9 ± 3.9	0.84
LVESV (mL)	0.2 ± 0.0	0.2 ± 0.0	0.3 ± 0.0	0.5 ± 0.1	0.21
LVEDV (mL)	0.8 ± 0.1	1.0 ± 0.0	1.1 ± 0.1	1.4 ± 0.2	0.04*
E:A	1.9 ± 0.1	2.0 ± 0.1	1.8 ± 0.1	2.0 ± 0.1	0.08
E vel (cm/s)	1.9 ± 0.1	54.3 ± 2.2	55.1 ± 2.3	57.1 ± 3.0	0.52
A vel (cm/s)	1.9 ± 0.1	27.4 ± 1.5	31.7 ± 2.1	29.2 ± 2.3	0.11

Data are expressed ± SEM

*nDM* nondiabetic, *DM* type 2 diabetic ZDF rats, *LVPWs* left ventricle posterior wall thickness systole, *LVPWd* left ventricle posterior wall thickness diastole, *EF* ejection fraction, *LVESV* end systolic volume, *LVEDV* end diastolic volume

For comparison of condition effect between nDM and DM at 12 and 20 weeks of age in all parameters two between groups ANOVA, * p < 0.05

**Fig. 2:**
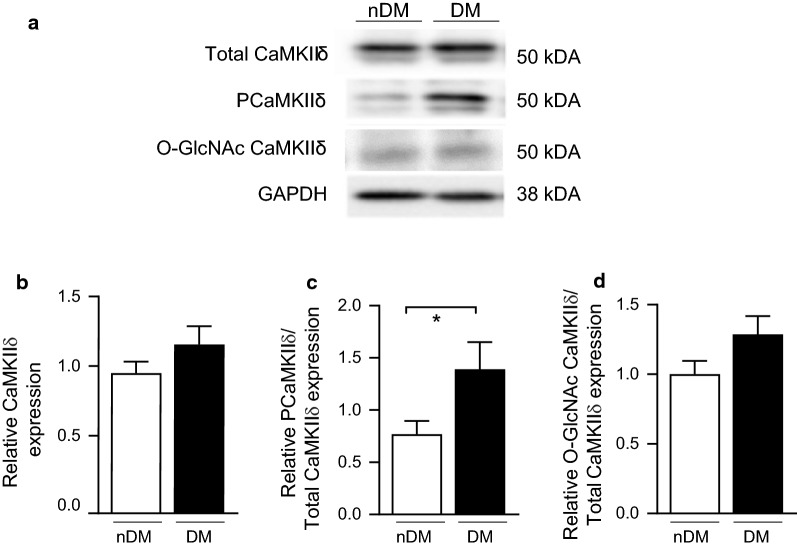
CaMKIIδ activation is increased in right ventricular tissue from type 2 diabetic ZDF rats compared to non-diabetic controls. **a** Representative immunoblot of total CaMKIIδ, Thr287 phosphorylated (PCaMKIIδ) and GAPDH as a loading control in nDM and DM ZDF right ventricle tissue. **b** Quantification of total CaMKIIδ levels in nDM (n = 8) and DM (n = 8) ZDF right ventricle tissue. **c** Quantification of PCaMKIIδ levels in nDM (n = 8) and DM rats (n = 8). **d** Quantification of O-GlcNAc modified CaMKIIδ levels in non-diabetic nDM and DM ZDF right ventricular tissue (DM) (n = 5). Data are mean ± SEM. *p < 0.05 for comparison of nDM and DM values (unpaired t-test)

Having established the ZDF rat model to parallel our patient cohorts, we subsequently measured CaMKIIδ expression and modification at 20 weeks in tissue from the RV (Fig. 2a). Similar to our finding in the human RAA samples, total CaMKIIδ expression was unchanged in the hearts of DM ZDF rats compared to lean controls (0.95 ± 0.08 vs. 1.15 ± 0.1, nDM vs. type 2 DM; Fig. 2b), and CaMKIIδ phosphorylation was significantly increased in the DM animals (0.77 ± 0.13 vs. 1.40 ± 0.26, non-diabetes vs. type 2 diabetes; Fig. 2c). We also observed an increase in O-GlcNAc modified CaMKIIδ in the DM animals (1.00 ± 0.19 vs. 1.38 ± 0.25, nDM vs. DM; Fig. 2d), but this difference was not significant (p = 0.13). Overall, we find that enhanced cardiac CaMKIIδ activity precedes advanced heart failure both in DM humans and the DM ZDF rat model.

### Basal cardiac contractility is impaired in DM ZDF rats compared to nDM controls

**Fig. 3:**
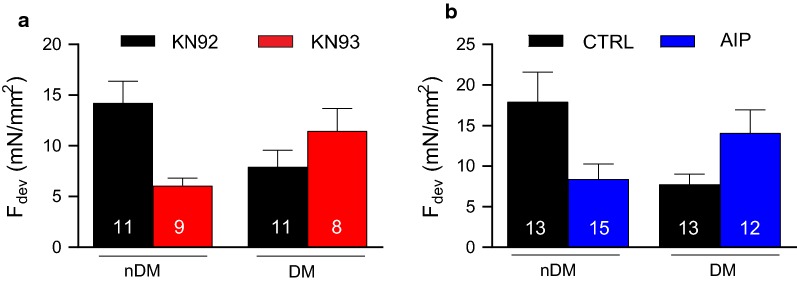
Developed force (F_dev_) at 2 Hz stimulation frequency is reduced in trabeculae from DM rats compared to nDM controls for trabeculae from nDM to DM rats. This effect is ablated by CaMKII inhibition via KN93 (**a**) or AIP (**b**). Data are mean ± SEM. n = 8–10 muscles per group. *p < 0.05 for comparison vs nDM KN92 (**a**) or nDM Control (**b**), two-way ANOVA

Diabetic cardiomyopathy is associated with reduced pressure development, which often leads to pathological hypertrophy [34]. We therefore hypothesized that basal contractility would be impaired in cardiac tissue from DM ZDF rats. Further, we hypothesized that our observed alterations in the activation of CaMKIIδ (Fig. 2), a potent regulator of cardiac proteins associated with contraction and relaxation, might underlie diabetes-induced impairment of contractile function. RV trabeculae from DM ZDF animals showed reduced developed force (F_dev_) at 2 Hz stimulation frequency compared to muscles from nDM animals, both in the presence of KN92, a control agent with no kinase inhibiting properties (p = 0.03, Fig. 3a), and in untreated controls (p = 0.02, Fig. 3b). These data suggest that, even at a relatively low pacing frequency, basal contractility and relaxation is impaired in the hearts of the DM ZDF rats.

Inhibition of CaMKIIδ has been demonstrated to rescue cardiac function in a variety of studies [17, 27, 35, 36]. We therefore hypothesized that inhibition of CaMKIIδ would reverse diabetes-induced alterations to contractility. Treatment with either KN93 or AIP, a pair of CaMKIIδ inhibitors, restored F_dev_ of trabeculae isolated from DM ZDF rats to levels comparable with nDM control trabeculae (Fig. 3a, b). Interestingly, inhibition of CaMKIIδ with either inhibitor reduced F_dev_ in the healthy nDM control trabeculae, consistent with prior reports that CaMKIIδ functions as an enhancer of contraction and relaxation in the heart. Thus, our data indicate that, even at a low pacing frequency, CaMKIIδ inhibition improves contractile performance in trabeculae from DM (but not nDM) rats.

### CaMKIIδ inhibition with KN93 partially restores cardiac contractility in trabeculae from DM ZDF rats

**Fig. 4:**
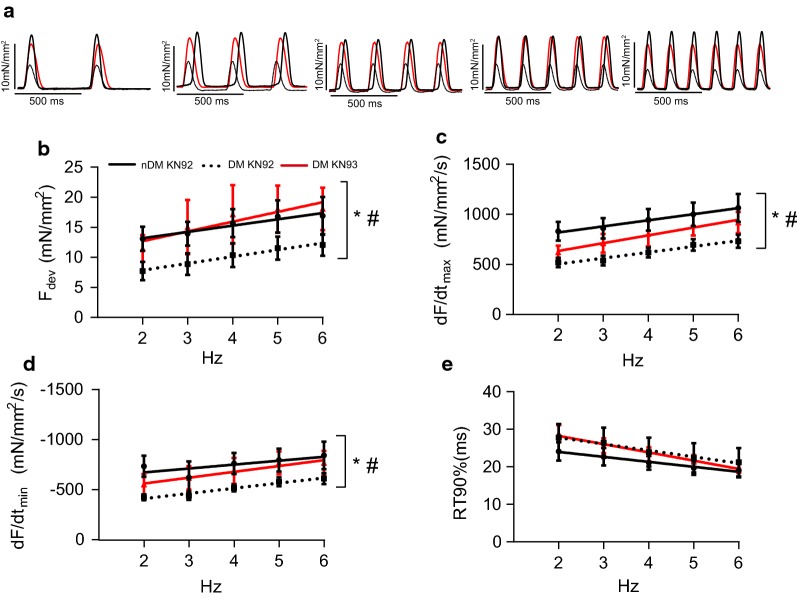
The CaMKII inhibitor KN93 restores developed force and partially restores rates of contraction and relaxation in trabeculae from ZDF rats. **a** Representative single twitches in the type 2 diabetic ZDF rat trabeculae in the presence of KN92 (straight black line) or KN93 (straight red line) at different stimulation frequencies. **b** Developed force (F_dev_). **c** Maximum rate of contraction (dF/dt_max_). **d** Maximum rate of relaxation (dF/dt_min_). **e** Relaxation time from 50 to 90% relaxation (RT90%). Data are mean ± SEM. Non-diabetic ZDF rat KN92, dotted line, n = 9; Type 2 diabetic ZDF rat KN92, black straight line, n = 9; Type 2 diabetic ZDF rat KN93, red straight line, n = 7. Data are mean ± SEM. For all parameters two-way between groups ANOVA: condition effect, ^#^p < 0.05 and treatment effect (KN93) *p < 0.05

To further clarify the effects of CaMKIIδ inhibition on contractility in the DM myocardium, we subsequently investigated the role of CaMKIIδ in modulating both contraction and relaxation over a range of stimulation frequencies (2–6 Hz) using the CaMKIIδ inhibitor KN93 (Fig. 4). To control for any potential off-target effects of KN93, all trabeculae were also tested in the presence of the control compound KN92.

Across the range of stimulation frequencies tested, we observed a significant reduction in absolute F_dev_ in trabeculae from DM rats treated with KN92 compared to lean controls (p = 0.01 for condition effect, Fig. 4b). The positive correlation between stimulation frequency and F_dev_ was unaltered in the DM tissue, suggesting that the diabetic myocardium has impaired force production rather than reduced responsiveness to stimulation frequency. We observed a trend towards prolonged time to reach 90% peak force (RT90%) in DM ZDF trabeculae, but the condition effect was not significant (p = 0.19, Fig. 4e). Conversely, both positive (dF/dt_max_) and negative peak derivative forces (dF/dt_min_) were significantly reduced in the trabeculae from DM ZDF rats (p < 0.0001 for condition effect, Fig. 4c, d), demonstrating that both contraction and relaxation are severely impaired in the diabetic myocardium. Treatment with KN93 fully restored F_dev_ across the entire range of stimulation frequencies tested to levels observed in trabeculae from nDM animals (p = 0.02 for treatment effect vs. diabetic + KN92, Fig. 4b). While KN93 treatment had no effect on RT90% (p = 0.45, Fig. 4e), it partially restored dF/dt_max_ and dF/dt_min_ (p = 0.01 for treatment effect vs. diabetic + KN92, Fig. 4c, d, respectively). Thus, CaMKIIδ inhibition using KN93 reversed the impaired contractility and partially reversed impaired relaxation in cardiac muscle from DM animals.

### CaMKIIδ inhibition with AIP fully restores cardiac contractility and partially ablates contractile frequency dependence in trabeculae from ZDF rats

**Fig. 5:**
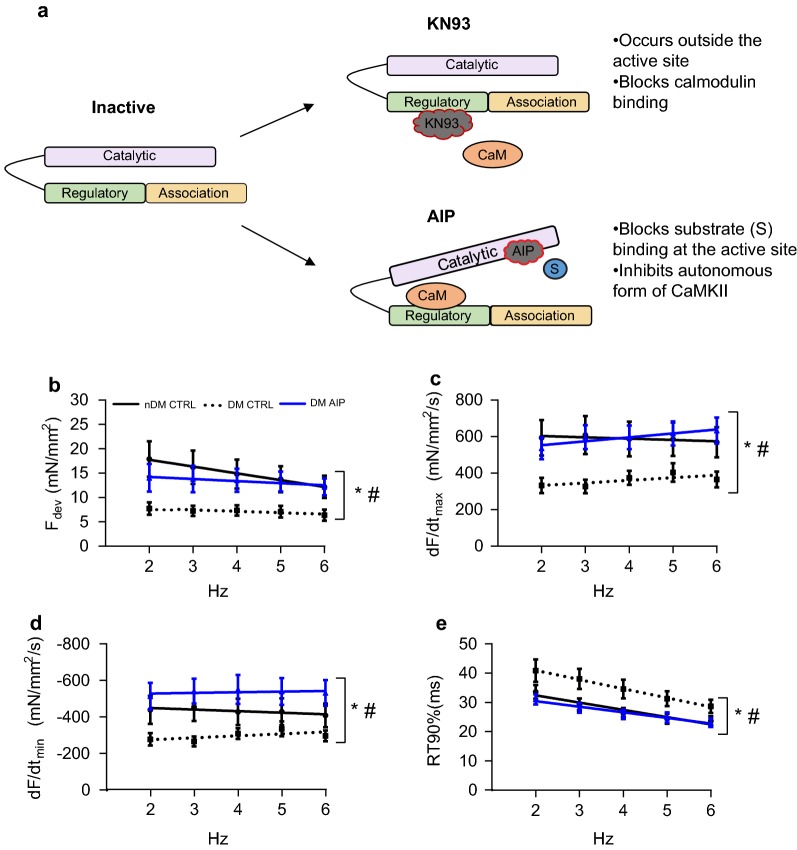
The CaMKII inhibitor AIP fully restores developed force and rates of contraction and relaxation in trabeculae from ZDF rats. **a** KN93 inhibits CaMKIIδ by preventing calmodulin binding at the regulatory domain, whereas AIP inhibits substrate binding at the catalytic domain. **b** Developed force (F_dev_). **c** Maximum rate of contraction (dF/dt_max_). **d** Maximum rate of relaxation (dF/dt_min_). **e** Relaxation time from 50 to 90% relaxation (RT90%). Data are mean ± SEM. Non-diabetic ZDF rat control, black straight line, n = 5, Type 2 diabetic ZDF rat control, dotted black line, n = 6; Type 2 diabetic ZDF rat AIP, blue straight line, n = 9. Data are mean ± SEM. For all parameters two-way between groups ANOVA: condition effect, ^#^p < 0.05 and treatment effect (AIP) *p < 0.05

KN93 inhibits CaMKIIδ activity by preventing calmodulin-binding to the kinase (Fig. 5a), but does not inhibit autonomously active CaMKIIδ [37]. Thus, we hypothesized that AIP, a peptide inhibitor of CaMKIIδ that blocks the catalytic domain and inhibits even the autonomously active form of the kinase, would be more effective at restoring cardiac function in trabeculae from DM animals. To this end, we directly determined the effects of AIP on trabeculae from DM ZDF rats by measuring contractility at 2–6 Hz (order of measurements with or without AIP was randomized). CaMKIIδ inhibition with AIP resulted in a significant increase in F_dev_ (p = 0.04), dF/dt_max_ (p = 0.02), and dF/dt_min_ (p = 0.04) across all frequencies (Fig. 5b–d). RT90% measured in DM trabeculae with and without AIP was not significantly different across the entire range of frequencies, (p = 0.08, Fig. 5e), but analysis using linear regression indicated that the relationship between RT90% and frequency was significantly altered after AIP treatment (p = 0.01).

Moreover, CaMKIIδ inhibition with AIP blunted the increase in contraction and the acceleration of relaxation in response to frequency across the range tested. The effects of CaMKIIδ inhibition were most pronounced at low frequencies, suggesting that a substantial fraction of total CaMKIIδ is active at baseline in the DM myocardium, consistent with our immunoblot data (Fig. 2b). Taken together, our data are consistent with the hypothesis that inhibition of CaMKIIδ improves cardiac function in the diabetic heart. Further, we have demonstrated that AIP, which inhibits all forms of CaMKIIδ activation, is more effective at restoring contractile function than KN93, which cannot inhibit autonomously active CaMKIIδ.

### Neither diabetes nor CaMKIIδ inhibition alters Ca^2+^ transient properties or SR Ca^2+^ load in rat myocytes

**Fig. 6:**
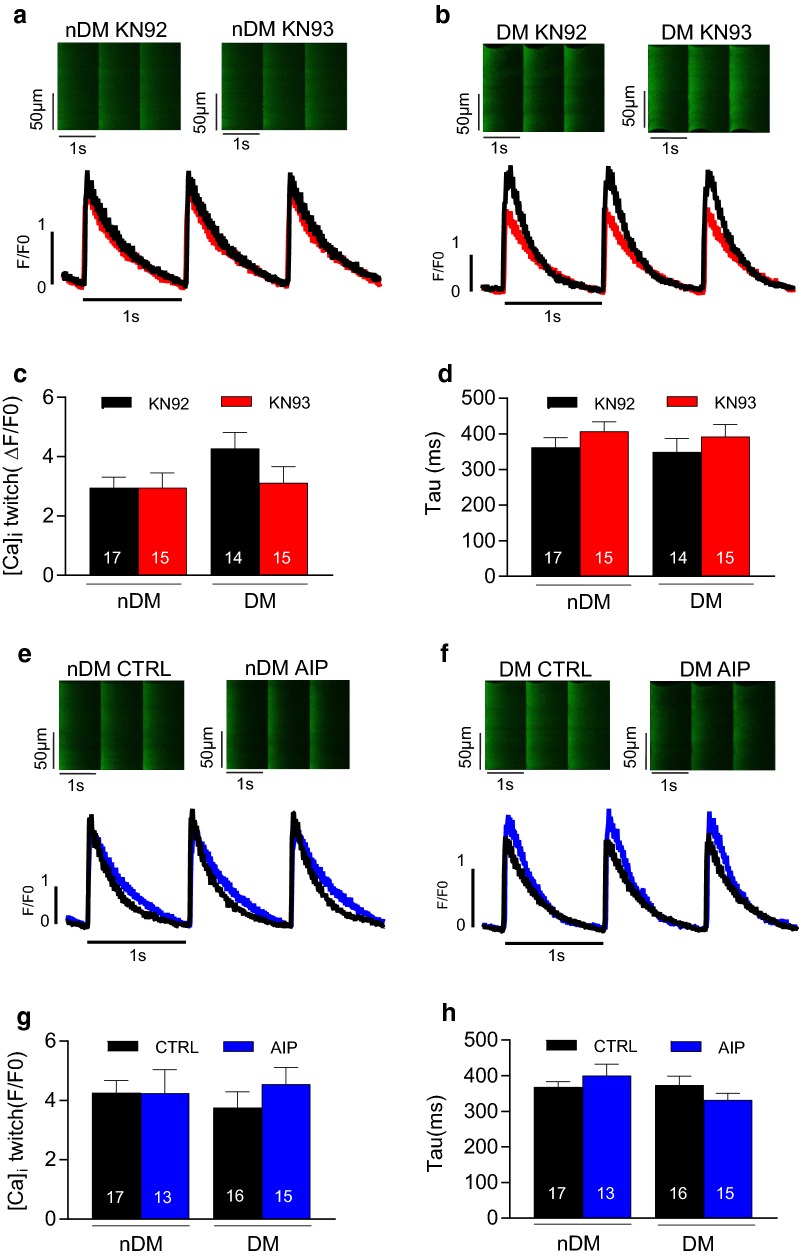
No effect of diabetes or CaMKIIδ inhibition with KN93 on Ca^2+^ transient properties. **a**, **b** Representative confocal line scan images (upper) and normalized Ca^2+^ transients (lower) from non-diabetic (nDM) and diabetic (DM) cardiomyocytes in the presence of KN92 and KN93. **c** Mean Ca^2+^ transient amplitude (F/F0, where F0 is diastolic fluorescence). **d** Mean time constant of Ca^2+^ decay (Tau). **e**, **f** Representative confocal line scan images (upper) and normalized Ca^2+^ transients (lower) from nDM to diabetic (DM) cardiomyocytes in the presence of CTRL and AIP. **g** Mean Ca^2+^ transient amplitude. **h** Mean time constant of Ca^2+^ decay (Tau). Data are mean ± SEM. n = 13–17 cells. For all parameters two-way between groups ANOVA: treatment effect *p < 0.05

Active CaMKIIδ can phosphorylate a number of proteins associated with Ca^2+^ flux in myocytes, including ryanodine receptors [12], phospholamban [13], and L-type Ca^2+^ channels [14]. We therefore hypothesized that diabetes impairs cardiac contractility by altering myocyte Ca^2+^ handling, and further, that inhibition of CaMKIIδ restores contractility by normalizing Ca^2+^ flux in myocytes. To test this hypothesis, we measured Ca^2+^ transients and SR Ca^2+^ load in isolated ventricular cardiomyocytes from 20 week old nDM and DM ZDF rats (Fig. 6). No significant difference was observed in the mean Ca^2+^ transient amplitude or tau (transient decay) between the nDM and DM cells in the presence of the KN92 control (Fig. 6c, d). Moreover, we found no effect of KN93 on Ca^2+^ transient amplitude or tau in either the nDM or DM cells (Fig. 6c, d). Similarly, we observed no difference in transient amplitude or tau between the nDM and DM cells treated with control buffer, and the CaMKIIδ inhibitor AIP had no significant effect on transient properties (Fig. 6e–h).

**Fig. 7:**
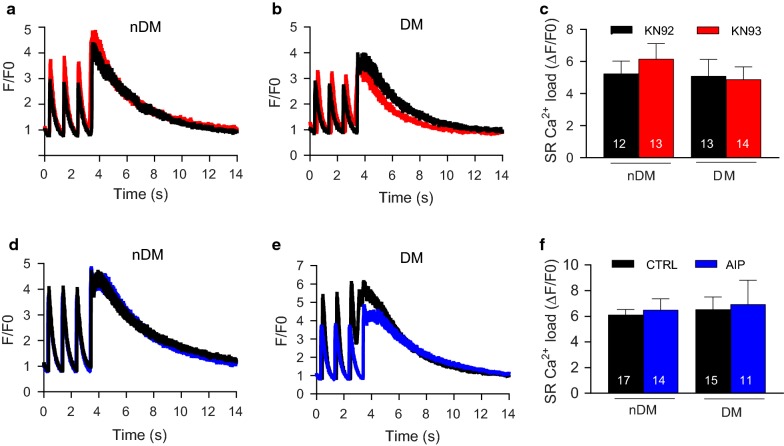
Sarcoplasmic reticulum (SR) content is not effected by diabetes or CaMKIIδ inhibition. **a**, **b** Representative normalized sarcoplasmic reticulum Ca^2+^ in nDM and DM KN92 (black line) and nDM KN93 (red line) treated cells. **c** Mean SR content assessed by 10 mmol/L caffeine-induced fluorescence change (Δ/F0) in nDM and DM KN92 and KN93 treated cells. **d**, **e** Representative normalized sarcoplasmic reticulum Ca^2+^ in nDM and DM control (black line) and AIP (blue line) treated cells. **f** Mean SR content assessed by 10 mmol/L caffeine-induced fluorescence change (Δ/F0) in nDM and DM control and AIP treated cells. Data are mean ± SEM. n = 11–17 cells. For all parameters two-way between groups ANOVA, p  ≤.05 was used

Our previous results indicated that reduced contractility observed in the diabetic trabeculae cannot be attributed to reduced Ca^2+^ release during transients. Therefore, we next tested the hypothesis that cardiomyocytes from DM ZDF rats have a reduction in Ca^2+^ store size, which would reduce the ability of the diabetic cell to produce rapid or forceful contractions. To measure SR Ca^2+^ content, caffeine was applied to the cells, causing a sudden release of Ca^2+^ from the SR and a rapid increase in intracellular Ca^2+^ concentration. No significant differences in SR Ca^2+^ load between the nDM and DM cardiomyocytes treated with KN92 were observed, nor did we find any effect of KN93 (Fig. 7a–c). Similar findings were observed in the CTRL and AIP conditions, with no difference found between the nDM and DM cells in CTRL conditions or any effect of CaMKIIδinhibition with AIP on SR Ca^2+^ load (Fig. 7d–f). Taken together, these results do not support our hypothesis that alterations to Ca^2+^ flux underlie the diabetes-induced reduction in cardiac contractility.

## Discussion

Activation of the multifunctional kinase CaMKIIδ has emerged as a key nodal point in the translation of cellular stresses into downstream alterations to cardiac physiology [5, 11, 38]. Recent publications have extended the role of CaMKIIδ into hyperglycemia and type 1 diabetes [28, 29]. Here, we show that CaMKIIδ plays a critical role in reduced cardiac contractility associated with type 2 diabetes. Critically, we show that CaMKIIδ activation is enhanced in type 2 diabetes and alters contraction and relaxation prior to the development of a heart failure phenotype. Thus, these findings suggest that altered metabolism in the diabetic heart activates CaMKIIδ and alters function at the myocyte level, eventually leading to the development of systolic and diastolic dysfunction.

Impaired contractility underpins reduced cardiac performance and contributes to enhanced susceptibility to heart failure in the diabetic myocardium [39]. Thus, one of the goals in the treatment of diabetes is targeting cellular pathways that modulate cardiac contractility. Here, we provide the first evidence that inhibition of CaMKIIδ restores both the force and rate of contraction in ventricular trabeculae from rats with type 2 diabetes. These findings are consistent with a previous study demonstrating that CaMKIIδ inhibition improves contractility in cardiac muscle during heart failure [27]. The previous study showed improvement in force generation and contraction, but not in relaxation parameters, following CaMKIIδ inhibition in trabeculae from patients with heart failure. Interestingly, our study showed a positive effect for CaMKIIδ inhibition on both contraction and relaxation parameters in the diabetic myocardium. This difference may be attributable to alterations in expression and/or activity of proteins associated with cardiac relaxation or alterations to myofilament function during diabetes and/or heart failure. For example, sarco/endoplasmic reticulum Ca^2+^-ATPase (SERCA) expression was reduced in heart failure patients [27], whereas Lamberts et al. [40] showed in diabetic patients with preserved EF that myocardial SERCA2a expression was increased and phospholamban expression was decreased.

An important finding of this study was that CaMKIIδ inhibition in healthy nDM rats resulted in reduced F_dev_ (Fig. 3), thus confirming the role of CaMKIIδ in the positive modulation of inotropy in the healthy heart. These findings mirror previously published observations that inhibition of CaMKIIδ in the healthy myocardium disrupts the beneficial effects of exercise on cardiac function [41]. In addition a study from Kemi et al. [42] investigating aerobic interval training in healthy mice also suggested that the improved inotropy and lusitropy they observed in cardiomyocytes after exercise training was due to an increase CaMKIIδ activation. Taken together, these studies point to an important and positive role for CaMKIIδ in the healthy heart, a role that becomes pathological during chronic cardiac stress as occurs in, for example, type 2 diabetes.

Another important finding from this study was the observation that AIP, which inhibits both calmodulin-dependent and autonomous CaMKIIδ activation, is more effective for rescuing cardiac function in diabetic tissue than KN93, which inhibits only calmodulin-dependent activation of CaMKIIδ. This study showed that AIP was able to restore F_dev_ and dF/dt_max_ in the diabetic trabeculae to similar levels as observed in the nDM trabeculae and even improve dF/dt_min_ to levels above that in the nDM. Therefore AIP was able to fully restore contraction and relaxation in the DM trabeculae. AIP inhibits CaMKIIδ by blocking substrate binding at the catalytic domain and thus inhibits the autonomously active form of CaMKIIδ that is frequently associated with cardiac pathology [43]. Prolonged Ca^2+^ elevations at high frequency are a hallmark of cardiac stress and result in autonomous activation of CaMKIIδ via auto-phosphorylation [9]. Moreover, CaMKIIδ activity is enhanced in diabetic models due to post-translational modification of the kinase [28, 29], while other post-translational modifications of CaMKIIδ are emerging [44]. Our data using the two inhibitors is consistent with these prior studies, as only the AIP would be expected to inhibit CaMKIIδ after post-translational modification. More work is necessary to delineate the roles of various modifications in CaMKIIδ-induced alterations to contraction and relaxation in the type 2 diabetic heart.

One of the most widely acknowledged roles of CaMKIIδ in the heart is modulation of Ca^2+^ flux [45–48]. Interestingly, our data indicate that neither the differences in contractile performance between nDM and DM trabeculae, nor the restoration of contractility by CaMKIIδ inhibition, are derived from alterations to Ca^2+^ transients or SR load. However, a number of alternative explanations remain unexplored. First, while baseline Ca^2+^ handling was not altered, these results do not preclude the possibility that spontaneous Ca^2+^ release events may be altering contractility in the diabetic myocardium. Indeed, CaMKIIδ inhibition has previously been shown to reduce Ca^2+^ sparks and ameliorate arrhythmogenic events in the diabetic heart [28]. Another potential target for alteration of cardiac contractility during diabetes is the myofilament itself. Previous studies have shown that diabetes triggers impaired function at the myofilament [49, 50], and CaMKIIδ is known to phosphorylate proteins associated with the myofilament [51]. Future work will need to focus on the mechanism by which diabetes reduces contractility.

Type 2 diabetes is a worldwide epidemic such that, by the year 2035, 592 million people are projected to suffer from the disease [52]. The landmark Framingham study established a now well-recognized link between diabetes and cardiovascular disease [53], which remains the leading cause of mortality in diabetic patients. Inhibition of CaMKIIδ has been proposed as an exciting new avenue in the treatment of cardiovascular disease. The data in this study provide novel evidence of CaMKIIδ activation as mediator of cardiac contractility in the type 2 diabetic heart and provide novel evidence and point to the potential for targeting CaMKIIδ as a therapeutic approach in diabetic patients to prevent structural remodeling and subsequent heart failure that arise from diabetes-induced impairment of contractile performance. Indeed, a new generation of CaMKIIδ inhibitors is currently in development that may have significant impact on the treatment of DCD and other forms of cardiovascular disease [43, 54]. In addition to pharmacological CaMKIIδ inhibitors, exercise is emerging as a therapeutic approach that may ablate the negative effects of CaMKIIδ in the diabetic heart [55]. The benefits of exercise for diabetic patients are well known, such as improving glycemic control, blood lipid profiles and cardiovascular function [56]. Interestingly, aerobic interval training in type 2 diabetic mice has been shown to reduce the activation of CaMKIIδ and improve cardiac function [57]. Therefore the combination of pharmacological CaMKIIδ inhibitors and exercise-based approaches in diabetic patients warrants further investigation.

In this study, we showed that CaMKIIδ phosphorylation and O-GlcNAc modification were increased in RAA samples from DM patients compared to those from nDM patients. While the use of human tissue is critical for establishing that the underlying mechanisms that drive CaMKIIδactivation are similar between DM ZDF rats and diabetic human patients, we must acknowledge a couple limitations of this study. First, the number of patients in each cohort was small (7 per group). While this group size was sufficiently large to see significant differences in CaMKIIδ modification, it is critical to consider that small human cohorts will have a great deal of heterogeneity. Second, all tissue was donated by patients undergoing coronary artery bypass graft surgery, and therefore all patients (DM and nDM) were not fully healthy. Importantly, we were still able to detect significant CaMKIIδ activation by phosphorylation and by *O*-GlcNAc modification in the diabetic patients that rose above the non-diabetic cohort, even with the presence of confounding health issues. Finally, we were not able to match the tissues used between the human and rat experiments, as we did not have access to RV human tissue. This limitation raises the possibility that there are heart chamber differences in our echo and Western blot data between the two species. The current literature regarding biochemical features of different heart chambers is split, with examples of differences in expression of proteins between chambers [58] and examples of no differences between chambers [59]. Critically, the alterations in phosphorylation and O-GlcNAc modification brought about by diabetes were similar in direction and magnitude for the RAA and LV, consistent with the hypothesis that CaMKIIδmodification is increased throughout the diabetic heart.

## Conclusions

Here, we show that cardiac tissue both from type 2 diabetic patients and from DM ZDF diabetic rats has elevated CaMKIIδ activation, independent of heart failure, compared to nDM controls. Moreover, we show that trabeculae from DM ZDF diabetic rats have reduced contraction and relaxation performance compared to trabeculae from nDM rats, indicating that the altered metabolic state of diabetes directly impacts contractile performance of cardiac muscle independent of nerve input, catecholaminergic effects, or other factors. Finally, we show that two different inhibitors of cardiac CaMKIIδ are both able to restore contractile performance of trabeculae from DM ZDF rats to levels similar to nDM controls. Our data demonstrate that activation of CaMKIIδ underlies reduced force development and impaired contractility in the diabetic heart. Further, it suggests that inhibition of CaMKIIδ is a potential clinical approach to prevent heart failure associated with type 2 diabetes.

## Abbreviations

A: late diastolic filling velocity
AIP: autocamtide-2-related-inhibitor peptide
ANOVA: analysis of variance
Ca^2+^: calcium
CaMKIIδ: calcium/calmodulin-dependent kinase II-delta
DCD: diabetic cardiac dysfunction
dF/dt_max_: positive peak derivative force
dF/dt_min_: negative peak derivative force
E: peak early diastolic filling velocity
E/A: ratio of peak early to late diastolic filling velocities
EF: ejection fraction
F_dev_: eveloped force
HbA1C: glycated hemoglobin
KHB: Krebs–Henseleit buffer
LV: left ventricular
LVEDV: left ventricular volumes at end-diastole
LVESV: left ventricular volumes at end-systole
RAA: right atrial appendages
RT90%: time to reach 90% peak force
RV: right ventricular
SERCA: sarco/endoplasmic reticulum Ca^2+^-ATPase
SR: sarcoplasmic reticulum
ZDF: Zucker Diabetic Fatty rats
